# RETRACTION: Administration of Melatonin in Diabetic Retinopathy Is Effective and Improves the Efficacy of Mesenchymal Stem Cell Treatment

**DOI:** 10.1155/sci/9763520

**Published:** 2026-04-17

**Authors:** Stem Cells International

RETRACTION: S. H. Abdel‐Kawi and K. S. Hashem, “Administration of Melatonin in Diabetic Retinopathy Is Effective and Improves the Efficacy of Mesenchymal Stem Cell Treatment,” *Stem Cells International* 2022, no. 1 (2022): 6342594, https://doi.org/10.1155/2022/6342594.

The above article, published online on 11 April 2022 in Wiley Online Library (wileyonlinelibrary.com), has been retracted by agreement between the journal Associate Editor, James Adjaye; and John Wiley & Sons Ltd. The retraction has been agreed due to concerns raised with the figures. Initially inconsistencies were identified with Figure 4 on PubPeer [1]. However, subsequent analysis by the publisher also found concerns in Figures 3–6. Specifically:

• In Figure 3e, the PRP4 blots appear identical to the APOA1 blots
• There are repeated elements within the panels in Figure 4a,d,h
• There are repeated elements within the panels in Figure 5b,c
• There are repeated elements within the panels in Figure 6a,c–e
• There are repeated elements between Figure 6c,e

After assessment of the author’s response to the concerns by the editorial board, the results and conclusions are no longer deemed reliable. Therefore, the article is retracted.

The authors did not respond to the retraction.

